# Workers expect basic social skills but limited autonomy from future robots – a qualitative interview study and taxonomy for robot social skills

**DOI:** 10.3389/frobt.2026.1815966

**Published:** 2026-06-17

**Authors:** Lukas Retzer, Cornelia Niessen

**Affiliations:** Chair of Work and Organizational Psychology, Friedrich-Alexander-University Erlangen-Nürnberg, Erlangen, Germany

**Keywords:** human-robot interaction, robot autonomy, service robots, social robots, social skills

## Abstract

Research has shown that robots are perceived negatively when they are less socially skilled or more autonomous than users expect them to be. However, little is known about worker expectations of robot social skills and autonomy as developments in the field have been mostly guided by technological progress without addressing future users’ perspectives. Building on the literature on social skills for robots and humans, we propose a taxonomy for robot social skills and investigate the extent to which this taxonomy is reflected in employees’ expectations of social robots in a qualitative interview study of 20 workers from the hospitality, manufacturing, and care industries. Our results show that workers are able to provide specific preferences when discussing future robot use in their workplace. We argue that some basic robot social skills can be broadly implemented and derive a provisional set of technical requirements. Results also suggest that a robot’s degree of autonomy needs to be finely tuned to the context and its task.

## 1 Introduction

Shortages of skilled workers are already affecting many industries and, according to demographic projections, are expected to intensify. An aging workforce combined with declining birth rates is predicted to result in millions of unfilled positions across nearly all sectors (Earl et al., 2017). The emergence and steady advancement of automation through robotics is thought to reduce the need for human labor and, although fears of growing economic inequality, reduced wages, and rising unemployment remain to be adressed (Gomes, 2024; Restrepo, 2024), has thus been widely presented as a promising response to this crisis (Kaplan and Haenlein, 2020). However, for industries such as hospitality, prototype manufacturing, and care, which require communication and physical interaction with customers, coworkers, or patients, present robots lack important social skills, i.e., capabilities to effectively navigate the intricacies of social interactions with humans (Irfan et al., 2025; Lindgren, 2025; Merz et al., 2024). Implementing such advanced robot social skills requires high levels of robot autonomy (Castro-González et al., 2011), i.e., the extent to which a robot can act without external control (Beer et al., 2014). Yet elevated autonomy may itself be challenging for users (Złotowski et al., 2017). In light of ongoing advancements toward increasingly socially intelligent robots and given that workers are most directly affected by robot design decisions (Nikolova et al., 2024), this study aims to examine workers’ expectations and preferences regarding robot social skills and autonomy.

Over the past decades, numerous robot social skills have been proposed, implemented, and empirically evaluated across a wide range of robotic platforms (Breazeal et al., 2016; Dautenhahn, 2007; Mahdi et al., 2022). For example, Mahdi and colleagues (2022) have identified 344 robot models from the literature, imbued with up to twelve different social capabilities. These social capabilities refer mainly to the modalities through which a robot can communicate and receive information, ranging from lights, body movements, and gestures to facial expressions and speech. Such a technical approach offers clear advantages, for example, in identifying the preferred communication modality for a certain setting (e.g., Klüber and Onnasch, 2022). However, it remains limited in that it mostly concerns itself with the *mode* of robot social behavior, defined by communication channels like speech or physical touch (Gasteiger et al., 2023), and less with user expectations regarding the *content* of it, such as empathy or conflict resolution (Babel et al., 2021). To broaden this focus, we propose extending the conceptualization of robot social skills beyond communicative channels to also encompass the substantive qualities of social interaction, thereby systematically integrating users’ expectations regarding what robots communicate, how they interpret social cues, and how they respond within socially embedded work contexts. (Heggestad et al., 2023; Ross et al., 2025).

Rarely have researchers and designers asked beforehand, what kinds of social interactions workers would prefer and what kinds of social skills they expect robots to possess to navigate those interactions effectively (Søraa et al., 2023). As long as these questions remain open, issues like “expectation gaps” between worker expectations and robot capabilities can arise (Kwon et al., 2016). Research has shown that interacting with a robot whose communicative behavior falls short of users’ expectations can be frustrating, confusing, and worrisome for users (Irfan et al., 2025). There is also some evidence that users trust and like a robot less when it does not conform to their expectations of cultural social norms regarding politeness or directness of speech (Gasteiger et al., 2023). We thus argue that it is beneficial for human-robot interaction in the work context, when robot social behaviors are finely tuned to worker expectations, which the present study aims to identify.

Particularly highly autonomous robots require well-developed social skills. However, empirical findings indicate that autonomous robots are not consistently evaluated positively by users (Naneva et al., 2020; for meta-analytic evidence; Ötting et al. (2022)). While some conceptualizations assume that greater robot autonomy entails reduced interaction, because the robot can execute tasks for extended periods without human supervision (e.g., Yanco and Drury, 2004), other approaches contend that high autonomy enables more frequent and more sophisticated forms of interaction (e.g., Feil-Seifer and Matarić, 2009). From the former perspective, robot autonomy may be framed as a loss of human control, which tends to be perceived negatively (Złotowski et al., 2017). From the latter, autonomy constitutes a prerequisite for effective task performance (Ötting et al., 2022) as well as for rich and engaging social interaction (Paluch et al., 2022). Against this backdrop, we additionally investigate how individuals from different industries respond to varying levels of robot autonomy, and how these responses are shaped by the robots’ social capabilities.

Research indicates that the relationship between robot characteristics and outcomes, such as trust and acceptance, depends on the industry and the tasks involved (Naneva et al., 2020; Onnasch and Hildebrandt, 2021; Pinto et al., 2025; Roesler et al., 2022). For instance, a human-like appearance is more commonly favored in the care sector than in the industrial sector (Klüber and Onnasch, 2022; Roesler et al., 2022) and educational robots are granted more autonomy than those dispensing medication (Feil-Seifer and Matarić, 2009). Building on this, we investigate the expectations employees in the sectors hospitality, prototype manufacturing, and care have regarding (1) social skills and (2) the autonomy granted to robots.

The aims of this paper are threefold. Firstly, we want to advance the current understanding of robot social skills by drawing on the literature on social skills in humans (Heggestad et al., 2023; Ross et al., 2025) and by providing a taxonomy to structure the resulting concepts. Secondly, we aim to test our taxonomy in an interview study with workers from different industries. Finally, we aim to address the challenge of identifying an optimal degree of autonomy in social human-robot interaction by observing the compromises workers intuitively make between their need for control and their expectations of efficiency.

## 2 Theoretical background

While the increasing augmentation and support of human work through robotization seems feasible, it is at this time hard to conceive of the total replacement of human labor by robots (Huang et al., 2019). At least in the foreseeable future, humans will have to work alongside and collaborate with robots in some way. Different patterns of interaction have been proposed by researchers and realized by designers, from human operation of the robot to true cooperation on a shared task (Krüger et al., 2009; Onnasch and Roesler, 2021). All of them have in common that the worker must directly interact with the robot as an agent that is increasingly different from a simple tool and at least partially intelligent and autonomous (Matheson et al., 2019). Several robot features and capabilities have been shown to shape these direct human-robot interactions (Ötting et al., 2022). While some factors rather unambiguously benefit workers, for example, clear interface feedback and adaptability to user needs (Ötting et al., 2022), the present research focusses on a selection of features, for which the evidence base is less straightforward, beginning with the robot’s social skills.

### 2.1 Robot social skills

Building on seminal work by Dautenhahn (2007), we assume a broad understanding of robot social skills as those capabilities which enable a robot to interact with and operate in a social environment. This goes beyond skills such as navigation around stationary obstacles and manipulation of workpieces which are necessary for the robot to interact with its non-social environment (Dautenhahn, 2007). It has long been argued that such social skills are necessary for robots to effectively interact and collaborate with humans (Breazeal et al., 2004; Dautenhahn, 2007; Fink, 2012). Consequently, the field of social robotics has spawned many prototypes imbued with a wide range of interactive capabilities (Breazeal et al., 2016; Mahdi et al., 2022; Niculescu et al., 2025). While there is no agreed upon definition of robot social skills, there is some overlap in current categorizations. For example, Breazeal and colleagues (2016) propose two sets of robot social skills (socio-cognitive skills such as shared attention and perspective taking, as well as communication skills such as verbal and non-verbal communication) in addition to the dimension of robot social-emotional intelligence (i.e., capabilities which contribute to the robot’s ability to recognize and display emotional cues). These three core social skills are mirrored in more recent work by Niculescu and colleagues (2025), who also suggest skills related to social cognition and communication, as well as emotional intelligence.

While this threefold division provides a useful starting point, current conceptualizations of robot social skills may nevertheless remain incomplete. Much of the existing literature concentrates on technological feasibility at a given stage of development, for example, advances in large language models (e.g., Wang et al., 2025) or neurocomputational approaches to emotion recognition (e.g., Spezialetti et al., 2020), without systematically considering how these capabilities align with users’ expectations of social interaction. To develop a more comprehensive perspective, we therefore turn to the literature on human social skills. This allows us to identify relevant conceptual dimensions that may have been underrepresented in the social robotics discourse, either because they are not yet central to currently envisioned application scenarios or because their technical realization has so far been limited.

The social skills framework proposed by Heggestad and colleagues (2023), as well as the conceptual clarification offered by Ross and colleagues (2025), structure a total of fifteen human social skills along the processual dynamics of social interaction. From this conceptualization, we included only those skills in our taxonomy which a robot could in principle be imbued with and accordingly excluded any skills that could not be translated into robot abilities, knowledge, or observable behavior (see Supplementary Appendix A; Table 9 for an extended overview of included and excluded concepts). First, we excluded three concepts, sociability, social self-efficacy, and impression management motives, which relate to unobservable inner states and presuppose inherently human experiences, beliefs, or desires. The personality trait of sociability is defined as the “tendency to experience ease in interpersonal relations” (Ross et al., 2025, p. 477) or, in other words, “how comfortable a person tends to feel in social situations” (Ross et al., 2025, p. 477). We do not believe that ease and comfort are qualities accessible to a robot and thus exclude this concept from our taxonomy. Furthermore, we exclude the two motivational concepts of social self-efficacy and impression management motives which are defined as “the belief in one’s ability to initiate and achieve social goals” (Ross et al., 2025, p. 477) and “the desire to cultivate a particular image” (Ross et al., 2025, p. 478), respectively. Such beliefs and desires are psychologically and physiologically complex motivational states informed by human qualities like emotional arousal and social persuasion (Anderson and Betz, 2001) which provide the thrust for the pursuit of social goals in humans (Heggestad et al., 2023; Ross et al., 2025). We acknowledge that some models employ the terms belief and desire in the context of robots and artificial intelligence. However, in these models, beliefs describe a system’s computational representations of the current state of the world informed by perceptual data and desires are representations of goal states the system currently strives to achieve (Georgeff et al., 1999). These terms are thus only loosely related to their original meaning in humans as they entail far less complexity and imply none of the motivational, emotional, and interpersonal correlates associated with the original terms. Additionally, they are unobservable dynamic system states and not stable qualities a robot can be designed to possess. As such, they are by definition beyond the scope of this work. We thus reduce the proposed core concepts from 15 to twelve. Additionally, the three concepts identified by Ross and colleagues (2025) as outcomes of social interactions are beyond the scope of this work as well, as they describe the results of an actor’s use of social behaviors and thus do not relate to capabilities or features which a robot could be imbued with. We are thus left with nine social skills concepts which relate to abilities, knowledge, or behaviors and are thus potentially relevant to robot design (see Table 1).

**TABLE 1:** A classification of robot social skill concepts.

Robot social skill categories	Robot social skill concepts	Examples from the three industries surveyed in this study
Socio-cognitive skills: The robot’s knowledge about social situations and its ability to perceive and interpret them	Social comprehension: The robot’s ability to perceive and understand human desires, feelings, and motives	Manufacturing: By perceiving an engineer’s facial expressions and utterances, the robot is able to understand that they are struggling with the weight of a part they’re holding. By analyzing their gaze, the robot can tell where the part is supposed to go
Communication skills: The robot’s ability to apply its socio-cognitive skills in the exchange of information and influence of others	Social awareness: The robot’s knowledge of how to change its self-presentation based on social context factors	Hospitality: The robot knows how to change its gestures and tone of voice to be perceived as friendly and polite by an elderly guest or as funny and exciting by a child
	Conversation skill^1^: The robot’s capability to use shared or expected communication conventions	Care: The robot is able to speed up its speech output when it’s being signaled urgency, to introduce itself when it meets a person it has not interacted with before, and to wave a hand and say goodbye at the end of an interaction
	Impression management tactics: The robot’s behavioral signals of self-referential characteristics	Manufacturing: While sweeping the shop floor, the robot explains with a smile on its display that it’s performing this task because it wants to be helpful for the team
	Persuasive messaging: The robot’s use of heuristic and/or reasoned communication tactics	Care: The robot employs a mixture of open-ended questions, health information, and active listening to encourage a patient to quit smoking
Socio-emotional skills: The robot’s capabilities to influence human emotional experiences and adapt its own emotional expression	Agreeableness: The robot’s capacity to elicit attributions of good naturedness, sympathy, tendermindedness, and flexibility	Hospitality: By listening attentively and adapting its voice to the emotional state it recognizes in a person, the robot is perceived as empathetic and warm by the restaurant guests it serves
Social problem-solving skills: The robot’s ability to perceive, define, and solve social problems and conflicts	Negotiation skill: The robot’s capability to align the needs or goals of multiple parties	Care: When one patient wants to watch TV and another wants to play a board game in the same room, the robot can perceive these positions, develop suggestions, and lead both patients to a compromise
	Exchange tactics: The robot’s use of incentives with interaction partners	Manufacturing: The robot encourages an employee to adhere to safety protocols by promising to bring them a cup of coffee
	Conflict management: The robot’s use of different levels of cooperative and/or assertive behaviors	Hospitality: On its way to deliver a tray of food, the robot does not allow itself to be persuaded by a child to play a game but instead offers to come back to them later

1 Originally named communication skill (Ross et al., 2025), we renamed this concept to avoid confusion with the higher order classification of communication skills.

To provide a coherent structure for our analysis, we classify the remaining nine robot-relevant social skill concepts into four overarching categories. Three categories—socio-cognitive skills, communication skills, and socio-emotional skills—are derived from prior robotics literature (Breazeal et al., 2016; Niculescu et al., 2025). We complement these with a fourth category, social problem-solving skills, following Grover et al. (2020). The resulting taxonomy is presented in Table 1. Our first research question is.

RQ1: Which social skills do workers expect robots in their workplace to possess?

### 2.2 Robot autonomy

When describing social human-robot interaction in the workplace, the robot’s autonomy, i.e., the “extent to which [it] can carry out its own operations and processes without external control” (Beer et al., 2014, p. 77), is one of the most fundamental aspects as it shapes the types, frequency, and intensity of interaction with the robot. Firstly, robot autonomy partly determines the worker’s role and the robot’s task by defining the robot as either equipment to be operated or a coworker to be cooperated with (Latikka et al., 2021). In other words, robots can be tools or partners, depending in part on their autonomy (Breazeal et al., 2004). Secondly, higher robot autonomy enables the worker to interact with the robot less frequently and neglect it for longer amounts of time (Beer et al., 2014; Yanco and Drury, 2004). A fully teleoperated robot needs to be interacted with during the entire task while a more autonomous model, like a delivery robot, can instead be supervised and given orders at regular intervals, between which it performs tasks without human oversight (Yanco and Drury, 2004). Thirdly, more intense interactions, such as those proposed for social robots, which go beyond merely mechanical tasks and include verbal communication and the expression and comprehension of emotional cues (Sarrica et al., 2020), necessitate higher degrees of robot autonomy in order for the robot to independently perceive and react to its social surroundings (Castro-González et al., 2011).

The impact of robot autonomy on human-robot interaction is thus manifold and complex. Some linear associations with outcomes of the interaction have been shown, e.g., improved performance and cognitive work-load (Ötting et al., 2022), but relationships with affective or motivational measures are likely more nuanced due to the “dilemma of [human-robot interaction] in the workplace” (Paluch et al., 2022, p. 381): While highly autonomous robots might be perceived as more helpful because they speed up tasks by requiring less human input (Ötting et al., 2022), they might also pose more of a threat to job security, physical safety, as well as human identity and distinctiveness (Złotowski et al., 2017). Robot autonomy as a design feature should therefore not be increased without a careful evaluation of the circumstances, including workers’ wishes and expectations.

In our understanding of robot autonomy, we build on the engineering psychology literature (Parasuraman et al., 2000; Wickens et al., 2021). Parasuraman and colleagues (2000) proposed four stages of interaction with automation (information acquisition, information analysis, decision selection, and action implementation), which can each be automated to different degrees, ranging on a continuum from low to high autonomy (Onnasch and Roesler, 2021). A seminal description of such a continuum of autonomy, which can be applied to robots with little adjustment (Beer et al., 2014), has been put forward by Sheridan and Verplank (1978). The authors describe ten levels of automation, ranging from level 1, where the human performs all decisions and actions without assistance, to level 10, where the automation acts fully autonomously without any human control. We attempt to identify and categorize worker expectations regarding robot autonomy in the same manner and posit our second research question:

RQ2: What are workers’ expectations and wishes towards robot autonomy in their workplace?

Most qualitative research on user preferences and expectations has focused on isolated industries like service (Paluch et al., 2022), manufacturing (Goštautaitė et al., 2024; Müller-Abdelrazeq et al., 2019; Welfare et al., 2019), which makes cross-industry comparisons impossible. We present results from all three of these industries in an attempt to show similarities and differences across different fields characterized by different kinds of tasks and challenges (Huang et al., 2019).

## 3 Methods

### 3.1 Participants

Between August and November 2024, we conducted 20 semi-structured qualitative interviews with employees from the hospitality (N = 5), prototype manufacturing (N = 5), and care industries (N = 10). On average, participants were 40.65 years old (*SD* = 13.05 years) and 70% (*n* = 14) were female (see Table 2). Participants had to be working in their respective industry for more than 1 year. We did not record further information about tenure.

**TABLE 2:** Sample characteristics.

Industry	Age		Gender distribution
	(*M* ± *SD*)	Range	
Hospitality (*n* = 5)	35.60 ± 11.52 years	22–50 years	♀ = 4; ♂ = 1
Manufacturing (*n* = 5)	45.80 ± 12.34 years	28–60 years	♀ = 1; ♂ = 4
Care (*n* = 10)	40.60 ± 14.28 years	27–67 years	♀ = 9; ♂ = 1
Total (*n* = 20)	40.65 ± 13.05 years	22–67 years	♀ = 14; ♂ = 6

The sample in care is twice the size of the other samples as it includes two branches (inpatient care as well as a combination of inpatient and outpatient settings) which have been subsumed under one category for this analysis.

Of the five participants from the hospitality industry, three were employees of a small cafeteria and two were employed at a medium sized restaurant and hotel. The cafeteria employed around 20 workers and served the employees of an assembly, sorting, and packaging workshop for the disabled. None of our interviewees themselves were disabled in relevant ways. There was practically no automation present in the cafeteria at the time of the interviews, and no plans present to automate tasks in the future. The restaurant and hotel employed about 90 employees and had partially automated certain tasks, e.g., through a self-service check-in counter, and implemented a robot vacuum cleaner to support the cleaning staff. There were no plans to implement robots to assist or replace wait staff, which our interviewees were a part of, in any way at the time of the interviews. Participants from the hospitality industry were about 5 years younger than the average participant (*M* = 35.60 years; *SD* = 11.52 years) and four of five were female.

All five interviewees from the manufacturing industry were employed at a medium-sized mechanical engineering firm, employing around 250 employees. They had acquired and implemented a welding robot as well as multiple CNC machines several years ago. All other tasks, such as logistics, surface treatment, and assembly were minimally automated, as the firm specialized in designing and building prototypes. Since they produced small quantities, broader automation was deemed unnecessary or even impossible. For this specific reason, one task, sheet metal processing, was still performed manually although the necessary equipment to partially automate it had been acquired. No workers had been laid off due to automation at this firm and none of our interviewees regularly worked with robots in any way. Participants from the manufacturing industry were on average 5 years older than the average participant (M = 45.80 years; SD = 12.34 years) and all but one of them were male.

Of the ten participants from the care industry we interviewed, five each were employed at two large care facilities, employing around 220 and 250 employees, respectively. The larger one of the two facilities only had permanent residents, the other one also offered outpatient services. The smaller facility had recently acquired and tested a mobile, articulated robot arm, but never successfully implemented it to augment or replace human labor in any way. It was not in use at the time of the interviews. No other significant efforts towards automation had taken place at either facility. On average, participants from the care industry were 40.60 years old (SD = 14.28 years) and nine of them were female.

Further sample characteristics are detailed in Table 2. None of the workers interviewed had relevant experience in working with robots. Interactions with robots in other places were also very limited. One worker from the care industry reported having been served by a service robot at a restaurant recently and described it as an interesting, novel experience. One worker from manufacturing stated he had been on tours of large automotive plants in the past and witnessed industrial robot lines there, which he described as impressive. No other prolonged robot interactions were reported in the sample.

Participants were first informed about the interview purpose and informed consent was obtained for them to be audio-recorded. We interviewed participants in their native language, which was German for all of them. Interviewees were given a brief overview of different robot applications in various fields in order to develop a mutual understanding and limit potential bias, as recommended by Klebbe and colleagues (2023). This was followed by the semi-structured interview. Participants received no compensation for taking part; however, they were able to participate during their working hours. All study data, including voice recordings, were pseudonymized using IDs and treated confidentially. In accordance with the recommendations by the German Research Foundation, this research required no formal ethics approval.

### 3.2 Topics of the semi-structured interviews

Four main topics were addressed in the interviews (see Supplementary Appendix B for complete interview guide): Daily tasks, possible robot applications, wishes and expectations, as well as fears regarding robots. The questions were phrased so they would not have to be changed for each industry.

The first set of questions aimed to provide insight into participants’ main daily tasks, including the tools, colleagues, and premises involved, the necessary qualifications, as well as hindrances, issues, and typical challenges which might arise during the tasks described. A sample question is “What exactly do you do in this task? What is the goal?”. With these questions we aimed to get an insight of participants’ daily experiences, actions, and situational factors. Building on reported challenges and daily hassles, the second part focused on possible robot applications in the interviewees’ workplaces. For each application the participants identified, they were asked to imagine the specific sequence of steps the task would consist of if it were assisted by a robot as well as potential problems they could anticipate. A sample question is “What would the exact sequence of the activity look like if it were performed by a robot?” This approach was intended to promote a more detailed visualization of human–robot interaction scenarios, providing a concrete context for discussing the main research questions, namely, expectations regarding robots’ social skills and desired levels of autonomy, in subsequent stages of the interview. This was expected to mitigate superficial general statements. Consequently, in the third part, questions aimed to identify participants’ wishes, expectations, and requirements of a robot if it were to collaborate with them in their workplace. This included questions about specific robot skills, features, and physical characteristics to implement or avoid, as well as the desired degree of robot autonomy. A sample question is “What would a robot absolutely have to be able to do in order to work in your field?” After participants had been imagining working with robots and possible consequences for themselves for some time, fourth, we asked questions about participants’ specific attitudes and fears towards robots as well as possible strategies to mitigate those fears. A sample question is “What are your greatest fears regarding the idea of having a robot in your company?” In a final question, we asked every participant to describe how they would describe the optimal human-robot-collaboration for their personal workplace.

### 3.3 Data analysis

The interviews ranged in duration from 16:55 to 57:21 min (*M* = 40:37 min; *SD* = 10:18 min). After completion, interviews were fully transcribed and coded in the original language.

**FIGURE 1:**
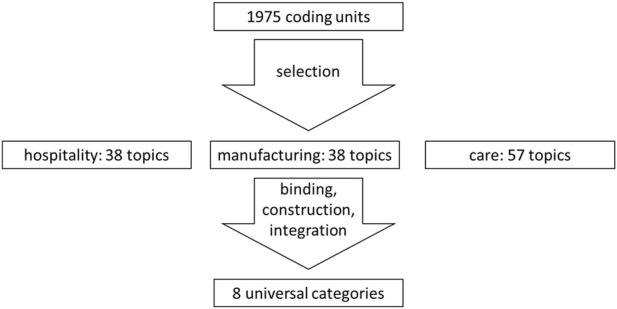
Flowchart of the summary content analysis.

We conducted summary content analysis according to Mayring (2014); Mayring and Fenzl (2019); (see Figure 1). In a first step, we identified 1975 relevant coding units, i.e., individual content-bearing phrases, ranging in length from a few words to several sentences, which relate to a singular topic, from the interviewees’ statements. An example is a quote from a participant in the hospitality industry:

*“I could imagine that a loud beeping sound when the thing is reversing or something like that could be a bit off-putting.”* (ID 2, hospitality).

The entire statement relates to one specific topic, i.e., design features, or more specifically, sounds to avoid, while the statements immediately before and after it related to other topics.

Then, we reduced the pool of coding units, first, inductively, i.e., guided by the texts, by means of selection, erasing semantically identical phrases, resulting in 38 unique topics for hospitality, 38 unique topics for manufacturing, and 57 unique topics for care. An example is the topic of *preferred manipulators* in manufacturing. Three different statements from two different participants were attributed to this topic:

*“And he can usually do that with two tongs, I would say, which are then organized with a camera system.”* (ID 7, manufacturing);

*“It would be better if he could grasp things. If one could say that he could also grasp something.”* (ID 9, manufacturing);

*“If he had to take a container from the shelf, then there would have to be something he could drive underneath it.”* (ID 9, manufacturing).

We performed a second, deductive reduction towards a higher level of abstraction by means of binding, construction, and integration, grouping the codes into the following eight categories, which largely correspond to sets of questions in the interview guide: Current tasks, currently challenging aspects of work, robot application scenarios, industry- or company-specific challenges for robot implementation, wishes and requirements for robot design, fears regarding robot implementation, attitudes towards robots, and potential advantages of implementing robots. Finally, we checked whether all original paraphrases were still represented by these eight categories.

For minimizing bias and ensuring data quality, 40% of the material (two interviews each in the hospitality and manufacturing industries as well as four of the interviews in the care industry) was coded by additional trained researchers (20% by one additional researcher and 20% by two additional researchers). To determine the inter-rater reliability, we computed Krippendorff’s Cu-Alpha in SPSS using the KALPHA macro by Hayes and Krippendorff (2007). This score can take values between 0 and 1, with higher values indicating higher reliability. The three researchers achieved a Cu-Alpha of 0.85, which is above the recommended threshold of 0.80 (Krippendorff, 2004).

## 4 Results

We first present the results of the summary content analysis separated by the categories identified in the last step of the analysis. These general results provide the basis for our attempt to answer the two main research questions: What are workers’ expectations of (1) robot social skills and (2) robot autonomy. Results regarding further robot characteristics and workers’ attitudes towards future collaboration with a robot, which served as background information, but are not the focus of the analysis, can be found in Supplementary Appendix C.

### 4.1 General results

Six categories identified from the interviews directly relate to robots. They are summarized, together with the respective topics, in Tables 4–8, Supplementary Appendix A.

*Robot application scenarios* (Table 3) include descriptions of specific scenarios of hypothetical robot use, including tasks a robot could perform and interactions that might result. When discussing potential applications of robots, most participants immediately identified *challenges for robot implementation* (Table 4), which we defined as aspects of their physical working environment, the organizational structure of the respective company or industry in which they work, psychosocial characteristics, or biomechanical aspects that must be considered when deploying robots or that could generally complicate their deployment. In order to deal with these challenges and fulfill the identified possible robot tasks, participants mentioned several *wishes and requirements* (Table 5) regarding the appearance, movement, communication, functions, or use of the hypothetical robot, including negative statements, i.e., aspects that are expressly not desired. The next category subsumes statements about *concerns* (Table 6) about the use of robots in the interviewee’s company or industry, both in terms of their own hypothetical interaction with the robot, its functions, or possible errors, and in terms of the consequences for their profession, the labor market, and society. In the next category, we collected statements about *attitudes* (Table 7) regarding robots, artificial intelligence, and modern technology in general, either held by the interviewees themselves or reported to be held by customers, colleagues, or patients, as well as possible future trajectories and means of influencing such attitudes. The final category collects statements made by the interviewees about *advantages* (Table 8) of robot use, i.e., positive consequences or aspects of work that would be improved by the hypothetical use of robots, either for themselves and their colleagues, or for other stakeholders in their respective industries.

**TABLE 3:** Possible robot applications identified in the interviews.

Robot application	Industry	Description
Cleaning	H, M, C	Maintenance of safety and hygienic standards
Errands	H, M, C	Fetching and delivering small items, documents, or parts
Heavy lifting	H, M, C	Vertical movement of objects or people
Information exchange	H, M, C	Enabling communication within the department and company, as well as with entities outside the organization
Reception	H, M, C	Greeting and providing information to guests at a point of entry
Dishes	H	Transport, serving, and cleaning of dishes
Taking orders	H	Eliciting and forwarding guests’ orders
Waste disposal	H	Collecting trash, emptying bins
Emotional support	H	Recognizing and calming down distressed employees
Employee training	H	Demonstrating specific tasks to trainees
Manipulation	M	Welding, milling, and edging, as well as the assembly of parts
Logistics	M	Packing, labeling, shipping, and receiving parts
Hazard avoidance	M	Remote-controlled solutions for potentially dangerous or difficult to reach areas
Team assistance	M	Assistance for meetings, project planning, or team coordination
Entertainment	C	Cognitively engaging patients
Mobilization	C	Physically engaging patients
Nutrition	C	Providing food and beverages
Documentation	C	Generating reports and photos as well as taking vitals
Safety patrol	C	Patrolling floors, reacting to emergencies, detecting damages, and accompanying staff
Translation	C	Providing, translating, and visualizing information during conversations
Laundry	C	Transporting and washing laundry, as well as making beds

**TABLE 4:** Reported challenges for robot implementation.

Challenge	Industry	Description
Spatial conditions	H, M, C	Spatial challenges for robot mobility
Unpredictability	H, M, C	High frequency of change regarding circumstances and requirements
Stakeholder diversity	H, C	The necessity of interacting with many different kinds of people
Precision	M	Working with small parts and valuable components
Legal conditions	C	Lack of clarity regarding legal issues
Health hazards	C	Significant potential for harm to human life

**TABLE 5:** Wishes and requirements for robot design and implementation.

Requirements	Industry	Description
Mobility	H, M, C	Effective movement around every part of the workspace
Safety	H, M, C	Protections against damage to people, the robot, or its environment
Communication	H, M, C	Features which enable effective vocal exchange of information
Appearance	H, M, C	Specific aesthetic features of the robot’s outer hull
Autonomy	H, M, C	Robot’s degree of independence in task selection and execution
Patience	H	Avoidance of additional time stressors
Ergonomics	H	Appropriate dimensions for physical interaction
Battery life	H	Uninterrupted usability during long shifts
Error tolerance	M	Flexibility in the face of user error
Manipulators	M	Appropriate tools for different part sizes and applications
Availability	M	Possibility to claim the robot’s assistance at any time
Self-cleaning	C	Automatic maintenance of hygienic standards on the robot itself
Sensors for vitals	C	Appropriate sensors for vitals like temperature, blood pressure*etc.*
Alarm function	C	Features which alert staff during emergencies
Voluntariness	C	Right to refuse working with the robot
Protocol	C	Reliable documentation of everything the robot did during a shift
Exchange	C	Opportunities to exchange experiences with other stakeholders

**TABLE 6:** Concerns regarding robotization and automation.

Concerns	Industry	Description
Collisions	H, M, C	Potential injuries or damage through collisions
Robot errors	H, M, C	Mistakes during task execution including issues of liability
Replacement	H, M, C	Negative impact on the labor market and employability
Skill loss	M, C	Negative long-term consequences for professional skill levels
Overreliance	M	Potential complete standstill of production in case of breakdowns
Societal impact	M	Possible negative effects of robots and AI on society at large
Privacy	C	Presence of cameras and other sensors in intimate situations
Dignity	C	Ethical concerns regarding robotic care for elderly or sick people
Patient aggression	C	Confusing effects on patients and possible subsequent aggression
Task load	C	Additional work caused by the robot

**TABLE 7:** Attitudes towards robot use in the workplace.

Attitude	Industry	Description
Openness	H, M, C	Curiosity, excitement, or interest towards robot use
Habituation	H, M, C	Increase in openness with time and amount of experience
Inevitability	H, M, C	Impossibility of avoiding robot use in the workplace in the future
Usefulness	H	Positive attitudes towards potential relief and support by robots
Appreciation	H	Robots as positive signals of worth by the employer
Integration	H	Expectations of robot integration into existing teams
Misallocation	C	Preferred investment toward job attractiveness, not robotization

**TABLE 8:** Potential advantages of robotization.

Advantage	Industry	Description
Saving time	H, M, C	More time to work on preferred tasks
Physical relief	H, M	Reduced strain, improved physical health
Cognitive relief	H, M	Focus on meaningful work, improved mental health
Staffing	H, C	Increased capacity to effectively deal with staffing shortages
Product quality	M	Less fluctuations in product quality due to human error
Cost reduction	M	Decreased costs for the organization (after amortization)
Learning	C	Opportunities to acquire new knowledge and skills from the robot
Companionship	C	Decreased loneliness
Coherence	C	Decreased intrusions and disturbances by daily hassles
Memory aid	C	Decreased risk of forgetting vital information
Understanding	C	Improved communication with people from other cultures and in foreign languages

### 4.2 RQ1: social skills

Building on these general results, we attempt to answer the first research question: What kinds of social skills do workers expect from robots in their workplace? We structure our results along the classification of robot social skills described above (see Table 1).

#### 4.2.1 Socio-cognitive skills

Building on frameworks for social skills in humans (Grover et al., 2020) as well as robots (Breazeal et al., 2016), we define socio-cognitive robot social skills as the robot’s knowledge about social situations and its ability to perceive and interpret them. The first specific skill from this category, participants reported in the interview is *social comprehension*, one of the core social skill concepts identified and defined by Ross and colleagues (2025) as the “ability to perceive and understand the desires, feelings, and motives of others” (p. 476). Social comprehension would thus enable a robot to recognize mental and emotional states in other social actors in its surroundings. Most participants in our sample expect robots in their workplace to be imbued with these perceptive powers:

“*Not only recognizing language and faces, but perhaps also recognizing the emotions behind them. Or recognizing how people are feeling at that moment.”* (ID 3, hospitality).

For the sake of initiating specific tasks, workers expect robots to be able to receive and interpret information, especially in the form of verbal commands:

“*Yes, firstly, I would probably have to call […] the robot. And, yes, I think it would be best to do it using a voice function […].”* (ID 11, care).

Some tasks, like entertaining patients, also require social comprehension during task execution:

“*Sometimes it's just important to have someone there who will listen. We have an elderly lady who also has dementia, and she's just happy when she can talk.”* (ID 18, care).

Beyond verbal input, expected social comprehension also encompasses the recognition of peoples’ needs and feelings in emergency situations:

*“When there is an alarm […]. First, just see what the resident needs.”* (ID 14, care).

As well as nonverbal signals in the context of pain assessment:

*“And there we also observe, for example, how they behave, their facial expressions, their gestures, and so on.”* (ID 11, care).

In addition to enabling specific tasks and forming a base for more complex skills, this ability to comprehend social surroundings is itself deemed necessary for safe and effective mobility and coexistence in the workplace, as robots are expected to be vigilant and avoid collisions:

*“When a robot is standing in the way and you need to go somewhere with the pallet truck. That would obviously be inconvenient for people because then they would have to take a detour.”* (ID 10, manufacturing).

#### 4.2.2 Communication skills

While social comprehension seems to be an important class of skills, workers also expect robots to be able to show communication skills, which, building on definitions from the human social skills literature (Grover et al., 2020; Ross et al., 2025), we define as a robot’s ability to apply its knowledge about social situations and translate it into action in the exchange of information and influence of others. We observe a large degree of agreement between participants in wanting the robot to be able to communicate and exchange information effectively and naturally:

*“Exactly, he should be able to talk to people. I think that's really important.”* (ID 3, hospitality).

The capability to visually, digitally, and verbally exchange information with people is deemed vital for a range of specific, communicative tasks such as guest reception and team assistance:

*“If a guest asks something like: ‘Where is the nearest pharmacy?’, then the screen displays: ‘You can find the nearest pharmacy […] in the shopping center […].’”* (ID 4, hospitality);

*“And you say [to the robot], ‘[…] I've quickly typed up what I want to discuss, and you go to the meeting for me.’”* (ID 7, manufacturing).

In addition to in-person communication, some participants also expect telecommunication capabilities to connect to people outside the organization:

*“He would also need to be able to make phone calls, because we get a lot of calls from people who need something.”* (ID 13, care).

Of the modes of communication discussed, worker expectations towards verbal exchange with robots are especially high, as they anticipate several issues such as overlapping voices, dialects and foreign languages, as well as people hard of hearing, which the robot needs to be able to adapt to:

*“Then the computer might be overwhelmed if someone else gives different commands, and things could go wrong.”* (ID 12, care);

*“[If] someone does not hear very well, there is still a button that they can use to turn up the volume. So that they can easily adjust it a little.”* (ID 3, hospitality).

The last part of our definition of communication skills goes beyond the exchange of information and includes behaviors which influence others and change their behavior in accordance with social goals (Grover et al., 2020). Related concepts include impression management tactics and persuasive messaging (Ross et al., 2025). We were unable to identify worker expectations towards such skills in our interviews, possibly because robots were not expected to have social goals of their own and instead only serve workers’ goals.

#### 4.2.3 Socio-emotional skills

When the robot is communicating with people, it will inevitably make an impression of some sort on the social actors around it. Socio-emotional skills, which, building on theorizing from the social robots (Breazeal et al., 2016; Niculescu et al., 2025) and human social skills (Grover et al., 2020) spheres, we define as the robot’s capabilities to influence human emotional experiences and adapt its own emotional expression, could contribute to more favorable impressions, allowing for the robot to be perceived as agreeable, i.e., “good-natured, sympathetic, tender-minded, and flexible” (McCrae and Costa, 1987; Ross et al., 2025).

According to our data, at least some workers expect robots to be able to improve emotional states in the people they interact with, specifically by expressing emotions themselves and adapting their emotional expression to the emotions displayed by others:

*“When he comes along and simply says a kind word and cheers [his coworkers] up, that's already a big help. […] That he can smile at you or empathize with you and say, ‘Yes, you're sad today. I understand you,’ and just have a sad face somehow.”* (ID 3, hospitality).

We emphasize that this skill goes beyond scripted polite robot behavior, as the adaptation to others’ emotional states entails the recognition and interpretation of affective signals from humans, as well as internal models of emotions informed by psychological theories of emotions in humans (Breazeal et al., 2016). This expectation was expressed most clearly in the context of specific tasks, such as coworker emotional support (hospitality) and patient entertainment (care), but not in manufacturing.

#### 4.2.4 Social problem-solving skills

Social interactions in the workplace do not always go smoothly and when conflicts or problems arise, the best solution is not always obvious (Babel et al., 2021). Grover and colleagues (2020) propose a set of skills which enable social actors to solve such problems by defining the problem, generating possible solutions, deciding which one to implement, and verifying implementation success. The literature on organizational behavior is also rich with related concepts, such as negotiation skill, exchange tactics, and conflict management (Ross et al., 2025). Social robotics research has shown that some conflict resolution strategies, such as humor and goal explanation, can be perceived positively, if robots apply them in a manner that is appropriate and sensitive to the context (Babel et al., 2021).

In our sample, none of these social problem-solving skills and behaviors were expected of robots in the workplace. This might be reflective of the tasks workers in our sample envisioned for robots in their organization, most of which involve human oversight. Social problem solving, much like emotional tasks, might implicitly be reserved for human workers.

In summary, robots are generally expected to be able to perceive, interpret, and adapt to social situations. In addition, workers expect to be able to communicate verbally with the robot in a fluent manner. For the two industries hospitality and care, which require empathetic interaction with guests or patients, robots are also expected to possess some basic socio-emotional skills. Other social skills, such as those pertaining to problem-solving, are not explicitly expected.

### 4.3 RQ2: robot autonomy

We see from these results that the amount of human control *versus* robot autonomy directly shapes possible tasks as well as expected social skills and behaviors. In general, we observe a large range of expectations and wishes towards robot autonomy, especially in manufacturing and care, ranging from the robot only acting upon explicit commands to fully autonomous robots acting with little to no oversight. Especially for the first three stages of automation, i.e., information acquisition, information analyses, and decision-making (Onnasch and Roesler, 2021; Parasuraman et al., 2000), expectations are spread across all possible levels of autonomy. Some participants, particularly in the care industry, where the average expected autonomy was lowest, believed that until a decision has been made and an explicit order has been given, the robot should remain inactive and offer no assistance, corresponding to level 1 [“The computer offers no assistance: human must take all decisions and actions” (Parasuraman et al., 2000, p. 287)] of Sheridan and Verplanck’s (1978) ten levels of autonomy:

*“I give orders, he has to respond, he has to report back, and then he gets the next order.”* (ID 12, care).

Towards the middle of these ten levels, where the robot may suggest actions but never execute them without human consent (level 5), some participants do expect a degree of independent thought from the robot, but still wish to remain in charge and have the final say:

*“He should think for himself, so to speak. But when I say something, he has to listen […]. So, a little bit of both.”* (ID 19, care).

Finally, a few participants believed robots would be able to offer the most support if they simply fulfill tasks autonomously without ever accosting the human, approaching the highest levels of automation:

*“The more autonomous, the better.”* (ID 10, manufacturing).

Expectations appeared less dispersed at the final stage of action implementation. Across manufacturing and care, participants tended to frame the control–autonomy dichotomy as a trade-off between safety and efficiency, favoring an intermediate level of both. This position aligned approximately with Level 8 of Sheridan and Verplank’s (1978) ten-level autonomy framework, in which the robot executes actions automatically once a decision has been made and informs the human only if requested.

*“Limits must be set […] in terms of system technology. […] Okay, you have a program sequence. That's what you do. You may also take the initiative yourself in the program sequence or try to find solutions on your own. But everything has to stay within the box.”* (ID 7, manufacturing).

Even the strictest of participants thus allow for some robot autonomy during action implementation, so long as the human remains in charge of decision selection.

Some participants presented a different form of compromise in the form of adaptive or adjustable automation (Feil-Seifer and Matarić, 2009; Parasuraman, 2000), where a robot would start off with low autonomy and gradually have it increased with growing experience and knowledge:

*“And at the beginning, it would be important for me that humans are always involved. […] But then, once you've convinced yourself, “That was great. That's okay,” the robot gains more and more trust, so to speak.”* (ID 8, manufacturing).

In hospitality, the issue of robot autonomy was debated less and there were no statements on the extreme ends of the control–autonomy spectrum. Instead, when the issue was raised at all, workers in this industry focused on true cooperation with the robot while maintaining a safe amount of human authority:

*“And, of course, he also needs to know who can give him an answer and who cannot. […] I'm not competition, but that I'm doing this together with him.”* (ID 3, hospitality).

In summary, while workers in manufacturing and care disagree how autonomously a robot should acquire and analyze information, most of our participants expect high, but not unlimited, robot autonomy during action implementation.

## 5 Discussion

This qualitative interview study set out to contribute to the literature on human-robot interaction by providing the perspectives of workers in three industries which are only now starting to be roboticized. We focused on workers’ expectations towards robot social skills and robot autonomy and specifically aimed to provide industry comparisons across the examined fields of hospitality, prototype manufacturing, and care. We show that workers are able to provide specific preferences when discussing future robot use in their workplace which we suggest can inform early design steps.

Our results illustrate a general openness and curiosity towards working with robots across a broad range of potential applications. As had previously been shown in the literature on social robots (Naneva et al., 2020), workers in our sample are largely willing to embrace useful, supportive systems and expect other stakeholders in their organizations to feel the same. One central prerequisite is a set of robot social skills, consisting of the abilities to perceive and interpret social situations, communicate verbally with social actors around them, and adapt to their emotional and cognitive states. Expectations are highest towards verbal communication; from the basic ability to receive and understand verbal commands to more advanced nuances of communication, such as emotional expression, workers expect to be able to interact with the robot in much the same way as they intuitively would with another human. However, there are also some socio-emotional and social problem-solving skills which our participants do not expect or do not want robots to possess, as they want emotional tasks to remain human responsibility and do not intuit robots to be able to navigate situations of conflict or negotiation.

Expected social skills seem to be closely tied to expected robot tasks in our data. Huang and colleagues (2019) proposed three types of tasks for automation, mechanical, thinking, and feeling tasks, along which we attempt to structure our results. In our study, *mechanical* tasks like the handling and moving of objects, only require socio-cognitive skills like the perception of people to avoid collisions or the capability to receive verbal commands. *Thinking* tasks such as analyzing information and providing advice, according to our results, additionally require communication skills to exchange information. Finally, *feeling* tasks like communication with people outside of the organization or caring for others, which in our sample were realized only in hospitality and care, also require the robot to be able to display emotional expressions and, at least to a very basic degree, influence the emotions of people around it. We also note that some of the tasks in the *feeling* category, like resolving conflicts or negotiating with others, would require a fourth set of robot social skills, i.e., social problem-solving skills. However, such tasks were not explicitly expected to be taken on by robots in our sample.

In addition, we report robot autonomy as an area of large disagreements between participants. As expected from earlier research (Ötting et al., 2022), workers across all three industries differed regarding the degrees of autonomy they would prefer. We interpret these findings along the theorizing by Paluch and colleagues (2022), who proposed that workers’ willingness to work with a service robot is the result of an appraisal process, weighing potential benefits and risks. Features like robot mobility, safety, and communication are uncontroversial, as they are clearly associated with increased benefits and decreased or stable risks. However, when robots are more autonomous, they are potentially more useful and at the same time perceived as more threatening: They might take on more tasks and fulfil them more effectively, however, the harm they could potentially cause when they make errors or replace human workers as well as the threat they pose to human distinctiveness and identity is increasing simultaneously (Ferrari et al., 2016; Złotowski et al., 2017). Workers’ preferences regarding autonomy might then depend on their individual characteristics, experiences, and biases which inform these appraisals, leading to a broader range of preferences. Future research needs to identify the specific characteristics of workers, tasks, and organizations which determine how robot autonomy is perceived.

Regarding the comparison of industries, we note several additional subtleties to be considered. Firstly, while basic social skills like social comprehension and verbal communication were expected across all three industries, more complex abilities involving the adaptation to emotions in humans were only discussed in hospitality and care, where feeling tasks (Huang et al., 2019) are more ubiquitous. Participants from the manufacturing industry on the other hand had lower expectations of robot social skills, likely informed by the types of tasks to be automated in this industry.

Secondly, we conclude that robot applications in the care industry need to be designed particularly carefully. Workers in care expressed the lowest expected degree of robot autonomy, indicating considerable hesitancy to give up human oversight and authority to automation. Additionally, the most severe worries regarding ethical considerations and privacy concerns were reported here and participants largely agree that some applications, specifically those that include medical and nursing care in the strictest sense, should be off limits.

In an effort to provide concrete takeaways for practitioners and robot designers, we further examine the most basic social skills, which we argue could be most relevant in hospitality, manufacturing, and care, and derive a provisional set of technical robot features they require. Social comprehension and social awareness require a broad range of capabilities on the robot’s part, including a theory of mind (Williams et al., 2022), recognition of speech, faces, emotions, and intentions, eye-tracking, as well as a deep knowledge of social and cultural norms. Conversation skill additionally requires speech production, optionally supported by gestures and other nonverbal output. For hospitality and care, the expected socio-emotional skills additionally necessitate emotional expression through paraverbal and nonverbal signals as well as more complex conversation strategies such as humor and empathy. We hope that future research will be able to exhaustively connect these and the remaining social skills we define in our taxonomy to specific technological requirements for robots, possibly in the form of a machine-readable ontology.

Regarding robot autonomy, the main takeaway we suggest is that stakeholders and specifically those who will have to collaborate with a robot should be able to participate in the design process, since optimal robot autonomy levels before and during decision-making likely depend on the precise setting and the personalities and experiences of the workers involved. We hope that this work has shown that workers will articulate quite concrete preferences when asked. When communicating with workers about robot autonomy, we have found it useful to frame the topic as a compromise between control with interruptions (low robot autonomy) and uninterrupted workflow (high robot autonomy). The question then becomes: how much control are workers willing to relinquish for decreased interruptions and increased efficiency? Options to adjust robot autonomy to individual preferences after implementation should be explored as well (Rabby et al., 2022). However, once the robot has received a task, we put forth a more specific suggestion. Here, optimal robot autonomy for our sample seems to sit around Level 8 of Sheridan and Verplank’s (1978) autonomy framework, where the robot executes its task autonomously and the user is only informed if they specifically request it.

When comparing these suggestions to the current state of robotics in hospitality and manufacturing, the contrast is stark. In hospitality, while some receptionist robots like the CPJROBOT PPBot report facial and speech recognition, as well as speech and gesture production and adjustable autonomy, the more ubiquitous serving robots like the Bear Robotics Servi + possess none of the capabilities we outline above. Similarly, in manufacturing, humanoid robots with some social capabilities, including speech recognition and production, by companies such as Figure AI or Agility are being tested for autonomous task execution in warehouse logistics and assembly. Most robots deployed in manufacturing settings, however, still closely resemble conventional industrial robots or cobots, where capabilities like intention and speech recognition are ongoing challenges and autonomy is limited (Taesi et al., 2023). Finally, in care, increasingly socially intelligent robots like the Care-O-Bot, which reports meeting most of the technical requirements derived above, including some socio-emotional skills, are implemented to alleviate patient loneliness and cognitively activate care recipients (Asgharian et al., 2022). Here, likely due to the importance of feeling tasks in this field, the gap between worker expectations and current robot abilities seems to be the smallest of the three industries we examined.

When interpreting these results, it is important to consider the sample of this study. While we can show some generalizability by interviewing participants from three different industries and broad age ranges, it has to be noted that we recruited a relatively small sample from a total of only five organizations in one country. In addition, due to the industries we chose to recruit from, none of our participants reported relevant prior robot exposure in the workplace. Expectations towards robots in more experienced workers or those with different cultural backgrounds might differ from those we observed. For example, experienced workers seem to have lower expectations of robot reliability and instead embrace occasional robot failures (Goštautaitė et al., 2024). Regarding cultural differences, Arabic speaking people have been shown to care more about robot politeness and apologizing, whereas Japanese people seem to prefer higher robot autonomy and interactions *via* touch-screen rather than speech (Gasteiger et al., 2023). This study is a first step towards identifying worker expectations and we encourage future research to expand on the specific scope chosen here.

This paper contributes to the human-robot interaction literature in several ways. Firstly, we advance the conceptualization of robot social skills beyond a narrow focus on communication modalities and sensor technologies by systematically integrating insights from the literature on human social skills (Heggestad et al., 2023; Ross et al., 2025). Building on this, we propose a taxonomy of robot social skills and, in this initial study, investigate the extent to which this taxonomy is reflected in employees’ expectations of social robots. In doing so, we respond to calls for user-centered qualitative research that can inform the design of social robots in workplace contexts across multiple industries (Baltrusch et al., 2022; Gasteiger et al., 2023; Søraa et al., 2023). Furthermore, we contribute to the ongoing debate on the appropriate level of social robot autonomy. Specifically, we address the challenge of identifying a degree of autonomy that is sufficiently high to enable meaningful social interaction (Feil-Seifer and Matarić, 2009), yet does not exceed workers’ expectations or undermine their sense of control (Gnambs and Appel, 2019; Latikka et al., 2021; Złotowski et al., 2017).

In summary, robot design features enabling basic social skills can be implemented broadly, while the topic of robot autonomy needs further research and will likely need to be finely tuned to the specific application and context. A set of robot social skills has emerged, consisting of socio-cognitive, communication, socio-emotional, and social problem-solving skills, which will likewise need further study.

## Data Availability

The original contributions presented in the study are included in the article/Supplementary Material, further inquiries can be directed to the corresponding author.
