# Supplementary material for: Workers expect basic social skills but limited autonomy from future robots – a qualitative interview study and taxonomy for robot social skills
Source: Front Robot AI. 2026 Jun 17;13:1815966. doi: 10.3389/frobt.2026.1815966 (PMC13318610; doi:10.3389/frobt.2026.1815966)
Supplement: Supplementary file 1 [file Supplementaryfile1.docx]

**Appendix A: Social skills concepts**

***Table 9.*** *Original list of social skills concepts identified by Ross et al. (2025) including reasons for inclusion or exclusion from our taxonomy*

| **Social skills concept (Ross et al., 2025)** | **Original definition** | **Included in taxonomy** | **Reasons for inclusion/ exclusion** |
| --- | --- | --- | --- |
| Social comprehension | The ability to perceive and understand the desires, feelings, and motives of others | Yes | Can be implemented in robots through appropriate sensors and algorithms |
| Agreeableness | The tendency to be good-natured, sympathetic, tender-minded, and flexible | Yes | Relates to the way an actor is perceived based on visible features and behaviors, which can be implemented in robots |
| Sociability | The tendency to experience ease in interpersonal relations | No | Unobservable inner state; experience of ease not implementable in robots |
| Social awareness | The knowledge of how to change one’s self-presentation based on social context factors | Yes | Can be implemented in robots through knowledge of social and cultural norms and expectations |
| Negotiation skill | The capability to align the needs or goals of multiple parties | Yes | Can be implemented in robots through combination of social comprehension and appropriate negotiation algorithms |
| Communication skill (renamed to conversation skill in our taxonomy) | The capability to use shared or expected communication conventions | Yes | Can be implemented in robots through knowledge of conventions and appropriate communication interfaces and channels |
| Social self-efficacy | The belief in one’s ability to initiate and achieve social goals | No | Unobservable inner state; human motivational states not implementable in robots |
| Impression management motives | The desire to cultivate a particular image | No | Unobservable inner state; human motivational states not implementable in robots |
| Impression management tactics | The behavioral signals of self-referential characteristics | Yes | Can be implemented in robots through appropriate behaviors and communication |
| Exchange tactics | The presentation of one or more incentives to an interaction partner | Yes | Can be implemented in robots as observable behaviors |
| Conflict management | The use of different levels of cooperative and/or assertive behaviors | Yes | Can be implemented in robots as observable behaviors |
| Persuasive messaging | The use of heuristic and/or reasoned communication tactics | Yes | Can be implemented in robots as observable behaviors |
| Elaboration likelihood | The degree to which an interaction partner processes a message systematically or heuristically | No | Interaction outcomes not implementable in robots |
| Social competence | The degree of goal attainment from an interaction episode or episodes | No | Interaction outcomes not implementable in robots |
| Social capital | The assets created from social relationships, such as trust, liking, and respect | No | Interaction outcomes not implementable in robots |
|  |  |  |  |

*Note*. See Table 1 for examples of hypothetical implementations of the concepts included in the taxonomy.
