# Supplementary material for: Workers expect basic social skills but limited autonomy from future robots – a qualitative interview study and taxonomy for robot social skills
Source: Front Robot AI. 2026 Jun 17;13:1815966. doi: 10.3389/frobt.2026.1815966 (PMC13318610; doi:10.3389/frobt.2026.1815966)
Supplement: Supplementary file 2 [file Supplementaryfile3.docx]

**Appendix C: Supplementary results**

In these additional results, we present workers’ expectations of robot appearance, as well as their attitudes which might shape reactions towards future collaboration with a robot.

**Robot appearance**

Apart from social skills and autonomy, which we report in our main manuscript, another feature which shapes human interaction with a robot is its appearance, specifically its degree of human likeness. In our interviews, there was a large range of wishes and opinions on this topic, especially in the hospitality and care industries. Some participants preferred a mechanical appearance:

*“I personally feel it would be better if it looked like a machine.”* (ID 2, hospitality),

while others insisted on an anthropomorphic design:

*“Yes, he must actually look like us, from a purely human perspective.”* (ID 12, care).

Most participants were trying to identify an optimal amount of human likeness which would enable effective interaction with it, not just for themselves, but also for guests or patients, without threatening human identity or resulting in confusion:

*“[Not] a real person, you can see it's still a robot, but still a little bit human.”* (ID 3, hospitality);

*“He really needs to be humanized a bit. Not like a mobile tower.”* (ID 15, care).

In addition to the dimension of human likeness, participants in the care industry voiced several priorities for robot design in their specific setting. Robots should look friendly, cute, or child-like, as well as noticeable and colorful, or at least not too dark. Some also mentioned zoomorphic preferences for robot appearance, as well as design cues from comic book characters or toys. These ideas were put forward mostly to further acceptance and perception among the sick and the elderly as well as avoid any connotation of darkness or death:

*“That [the residents] can easily recognize it.”* (ID 20, care);

*“Because people have to deal with death. [Black] might remind them of that […].”* (ID 19, care).

In manufacturing, robot appearance was discussed only insofar as it pertains to functional aspects. Here, participants reported no preferences either way on the spectrum of human likeness, so long as the robot is equipped to fulfil its duties:

*“I don't expect humanity from a robot. […] I would say it should serve its purpose. How it looks is irrelevant."* (ID 10, manufacturing).

**Attitudes towards robots in the workplace**

After discussing features of the robot which shape the human-robot interaction, we turn to the human side of it. In our interviews we wanted to capture participants’ attitudes and other relatively stable views to qualify and interpret findings from the previous research questions, as they had been previously shown to interact with wishes towards robot features like autonomy. Firstly, most participants reported a degree of openness, curiosity, excitement, or interest for themselves and most of the relevant stakeholders in their respective industries:

*“I'm not afraid of robots. I would also be happy if they came.”* (ID 3, hospitality);

*“I think that everyone would try it somehow.”* (ID 13, care).

The only qualifier offered across all industries was user age, where most participants agreed that openness would probably be lowest in higher age groups.

We also note a proportion of our sample, especially in care, who reported more hesitant or ambivalent attitudes towards robots, either for themselves or for other people at their workplace:

*“Well, we find that hard to imagine, because we have to adapt to new situations every day ourselves.”* (ID 12, care).

One reason provided for these stances might lie in feelings of lacking information and education on the topic:

*“I just don't know what a robot like that can do. I can't really imagine it.”* (ID 11, care).

Secondly, workers in the care industry seem to have found it harder to imagine significant potential benefits of robot implementation in their workplace. While interviewees from hospitality and manufacturing discussed the possible physical, psychological, and cognitive relief brough about by robots at length, there was no mention of this in the care industry. Instead, workers in care reported more concerns and fears surrounding robot use and robot errors. While the errors envisioned in the hospitality and manufacturing industries were somewhat harmless, like the mixing up of costumer orders or tools, participants in the care industry envisioned potential threats to human life after robot errors caused by internal or external issues:

*“That we ourselves might also be injured, knocked over.”* (ID 11, care);

*“Of course, I would be afraid of malfunctions. Like, if two wires somehow connect incorrectly and […] it starts burning […] and I can't control it anymore.”* (ID 15, care).

Another aspect of robot errors only mentioned in the care industry was the question of liability in case of an error:

*“Yes, who is responsible when something like that breaks down, […] because as a caregiver, you always have this underlying thought of ‘not doing anything wrong,’ because if something happens, you're responsible. That's why I think that's the biggest problem.”* (ID 19, care).

In addition, uniquely in care, participants mentioned feeling misunderstood as they faced a nursing staff shortage, which according to some of them, would have to be tackled by other investments in job attractiveness instead of robotization:

*“I'm talking about Germany now, the nursing crisis. I think there are better places to invest, places that are more interesting for us or that motivate other people, young people, to enter this profession.”* (ID 14, care).

Another regularly mentioned concern in the care industry was privacy, both of patients and workers, especially when envisioning robots with large numbers of cameras and other sensors:

*“Because I don't need to film anything in the bathroom […], and I don't need to film anything in bed either. If someone isn't feeling well.”* (ID 13, hospitality);

*“So filming, I think, is not okay. […] I would feel super controlled.”* (ID 11, care).

Broader ethical concerns regarding patient dignity were also discussed repeatedly:

*“Well, the interpersonal aspect a little bit. […] I don't think that's appropriate in this case. So, I would really have a guilty conscience about advocating that.”* (ID 13, care).

These views again point to an underlying feeling of uneasiness or ambivalence in the care industry and might explain why some interviewees remain skeptical of higher degrees of robot autonomy. Building on this issue, interviewees in all industries agreed that for those stakeholders who might be fearful or skeptical towards robots at first, habituation over time and with increasing positive experience would be possible:

*“You have to do it slowly to get rid of the fear in people.”* (ID 6, manufacturing);

*“If you do it anyway, then step by step you'll think, ‘Well, it's not so bad after all.’”* (ID 4, hospitality).

Another point of agreement we identified was the inevitability of robot use. Participants across all three industries mentioned the feeling that robots were going to be a part of their work life sooner or later, although they disagreed on the timeframe:

*“I always say, there's this great movie called ‘I, Robot.’ And something like that will happen someday. Maybe not in 10 years, but in 20-40 years, something like that will happen.”* (ID 6, manufacturing);

*“Let's talk about it again in a hundred years, shall we?”* (ID 12, care).

Finally, most of the interviewees had thought about the possible impact of robotization on the labor market. We note a high degree of insecurity and disagreement on this topic, with opinions covering a broad spectrum of positive and negative feelings:

*“The only thing I'm really worried about is jobs.”* (ID 6, manufacturing);

*“And I don't think it takes work away from our people, but rather that it can be an enrichment for them.”* (ID 3, hospitality).

Interestingly, referring back to the topic of robot appearance, some participants mentioned workers might feel their jobs were especially threatened by humanoid robots:

*“Because then it looks like a human being, and they think, ‘Okay, he's going to take my job away.’”* (ID 7, manufacturing).

Views such as this likely contribute to preferences for functional, non-anthropomorphic design.

Other attitudes mentioned specifically in the hospitality industry cover a range of mostly positive statements about the potential usefulness of robots, feeling appreciated by the employer when being provided with a robot helper, as well as the potential for quick integration of robots into teams. These positive views might explain why robot autonomy was less of a concern in hospitality and participants from this industry instead embraced cooperation with the robot.

In manufacturing, interviewees discussed some potential negative effects of robots and AI, for society at large as well as specific issues like skill development in their industry, which likely contribute to some of the sentiments prioritizing human control over robot autonomy in this field:

*“I still view it as a tool. It's not going to be my ‘best buddy’ or anything like that.”* (ID 10, manufacturing).
