# Supplementary material for: Workers expect basic social skills but limited autonomy from future robots – a qualitative interview study and taxonomy for robot social skills
Source: Front Robot AI. 2026 Jun 17;13:1815966. doi: 10.3389/frobt.2026.1815966 (PMC13318610; doi:10.3389/frobt.2026.1815966)
Supplement: Supplementary file 3 [file Supplementaryfile2.docx]

**Appendix B: Semi-structured interview-script**

German (see below for English translation)

1. Einführung
   1. Vorstellung des Interviewers und des Projektes
   2. Erklärung zum Ablauf des Interviews
   3. Einführung zum Thema „Roboter“ in verschiedenen Anwendungsbereichen (Industrie, Logistik, Dienstleistungen, Gesundheitssystem, Überwachung)
2. Tagesstruktur
   1. „Zuerst würden wir gerne etwas über Ihre Arbeitszeiten erfahren. Arbeiten Sie Vollzeit oder Teilzeit?“
   2. „Arbeiten Sie im Schichtsystem? Falls ja, welche Schichten arbeiten Sie normalerweise?“
3. Aufgaben
   1. „Bitte beschreiben Sie **einen typischen Arbeitstag**, Schritt für Schritt, ab dem Moment, an dem Sie Ihren Betrieb betreten. Schildern Sie bitte, was Sie im Laufe des Tages tun. Was sind Ihre Aufgaben im Laufe des Tages?“
      1. „Was tun Sie bei dieser Aufgabe konkret? Was ist das Ziel?“
      2. „Wie lange dauert diese Aufgabe? Wie beginnt sie und wie endet sie?“
      3. „Wo führen Sie die Aufgabe aus? Wie ist die Räumlichkeit beschaffen?“
      4. „Welche anderen Personen sind an der Aufgabe beteiligt oder aus anderen Gründen anwesend?“
      5. „Welche Werkzeuge/Hilfsmittel/Materialien/Maschinen nutzen Sie dabei?“
      6. „Welche Störfaktoren oder möglichen Problemquellen gibt es?“
   2. „Welche Ihrer aktuellen Aufgaben müssen **nicht unbedingt** von jemandem mit Ihrer Qualifikation gemacht werden? Wünschen Sie sich Unterstützung bei diesen Aufgaben?“
   3. „Welche Ihrer aktuellen Aufgaben müssen **unbedingt** von jemandem mit Ihrer Qualifikation gemacht werden? Wünschen Sie sich Unterstützung bei diesen Aufgaben?“
   4. „Unabhängig von Ihren Qualifikationen, gibt es Aufgaben, die für Sie besonders anstrengend sind – ob körperlich oder geistig?“
4. Mögliche Anwendungen
   1. „Können Sie sich vorstellen, dass ein Roboter Sie bei den gerade genannten Aufgaben unterstützt? In welchen der genannten Aufgaben sehen Sie eine gute Gelegenheit zum Einsatz von Robotern?“
   2. „Wie könnte der genaue Ablauf der Tätigkeit aussehen, wenn er von einem Roboter ausgeführt werden würde?“
   3. „Welche Probleme sehen Sie, wenn ein Roboter solche Tätigkeiten ausführen würde?“
5. Ansprüche & Erwartungen
   1. „Was müsste ein Roboter unbedingt können, um in Ihrem Bereich arbeiten zu können?“
   2. „Was wären No-Gos, die der Roboter in Ihrem Arbeitsbereich unbedingt vermeiden müsste? Können Sie sich vorstellen, dass der Roboter in Ihrem Arbeitsbereich völlig eigenständig arbeitet, ohne menschliche Begleitung?“
   3. „Wenn Sie an die Menschen denken, mit denen Sie arbeiten (Kolleg*innen, Kund*innen, Patient*innen, …), wie schätzen Sie deren Einstellung gegenüber neuen Technologien und besonders Robotern ein? Falls Ihre Einschätzung eher negativ ist, denken Sie, es ist möglich, dass sie sich an einen Roboter gewöhnen werden?“
   4. „Wenn Sie an die Menschen denken, mit denen Sie arbeiten (Kolleg*innen, Kund*innen, Patient*innen, …), was wären die größten Vorteile, die der Einsatz eines Roboters für sie hätte?“
6. Befürchtungen
   1. „Was sind Ihre größten Befürchtungen, die die Vorstellung von einem Roboter in Ihrem Betrieb bei Ihnen auslöst?“
   2. „Was müsste geschehen, um diesen Befürchtungen entgegenzuwirken?“
7. Abschluss: „Um das heutige Interview abzuschließen, würden wir Sie gerne noch bitte, uns Ihre Wunschvorstellung von zukünftiger Zusammenarbeit mit einem Roboter in Ihrem Betrieb zu beschreiben.“

Semi-structured interview-script – English translation

1. Introduction
   1. Introduction of the interviewer and the project
   2. Explanation of the interview process
   3. Introduction to the topic of “robots” in various areas of application (industry, logistics, services, healthcare, surveillance)
2. Daily structure
   1. "First, we would like to learn about your working hours. Do you work full-time or part-time?"
   2. “Do you work shifts? If so, what shifts do you normally work?”
3. Tasks
   1. “Please describe **a typical working day**, step by step, from the moment you enter your workplace. Please describe what you do during the day. What are your tasks during the day?”
      1. “What exactly do you do in this task? What is the goal?”
      2. “How long does this task take? How does it begin and how does it end?”
      3. “Where do you perform the task? What is the space like?”
      4. “What other people are involved in the task or present for other reasons?”
      5. “What tools/aids/materials/machines do you use?”
      6. “What disruptive factors or potential sources of problems are there?”
   2. “Which of your current tasks do **not necessarily** have to be performed by someone with your qualifications? Would you like assistance with these tasks?”
   3. “Which of your current tasks **absolutely must** be performed by someone with your qualifications? Would you like assistance with these tasks?”
   4. “Regardless of your qualifications, are there any tasks that you find particularly strenuous, either physically or mentally?”
4. Possible applications
   1. “Can you imagine a robot assisting you with the tasks you just mentioned? Which of the tasks mentioned do you see as a good opportunity for the use of robots?”
   2. “What would the exact sequence of the activity look like if it were performed by a robot?”
   3. “What problems do you see if a robot were to perform such activities?”
5. Requirements & expectations
   1. “What would a robot absolutely have to be able to do in order to work in your field?”
   2. “What would be no-gos that the robot would absolutely have to avoid in your field of work? Can you imagine the robot working completely independently in your field of work, without human supervision?”
   3. “When you think about the people you work with (colleagues, customers, patients, etc.), how would you assess their attitude toward new technologies, especially robots? If your assessment is rather negative, do you think it is possible that they will get used to a robot?”
   4. “When you think about the people you work with (colleagues, customers, patients, etc.), what would be the biggest advantages of using a robot for them?”
6. Fears
   1. “What are your greatest fears regarding the idea of having a robot in your company?”
   2. “What would need to happen to counteract these fears?”
7. Conclusion: “To conclude today's interview, we would like to ask you to describe your ideal scenario for future collaboration with a robot in your company.”
